# From Voxels to Physiology: A Review of Diffusion Magnetic Resonance Imaging Applications in Skeletal Muscle

**DOI:** 10.1002/jmri.29489

**Published:** 2024-06-20

**Authors:** David B. Berry, Joseph A. Gordon, Vincent Adair, Lawrence R. Frank, Samuel R. Ward

**Affiliations:** ^1^ Department of Orthopaedic Surgery University of California San Diego California USA; ^2^ Department of Medicine University of California San Diego California USA; ^3^ Center for Scientific Computation in Imaging University of California San Diego California USA; ^4^ Department of Radiology University of California San Diego California USA; ^5^ Department of Bioengineering University of California San Diego California USA

**Keywords:** diffusion tensor imaging, diffusion‐weighted imaging, skeletal muscle, muscle microstructure, muscle architecture, diffusion

## Abstract

Skeletal muscle has a classic structure function relationship; both skeletal muscle microstructure and architecture are directly related to force generating capacity. Biopsy, the gold standard for evaluating muscle microstructure, is highly invasive, destructive to muscle, and provides only a small amount of information about the entire volume of a muscle. Similarly, muscle fiber lengths and pennation angles, key features of muscle architecture predictive of muscle function, are traditionally studied via cadaveric dissection. Noninvasive techniques such as diffusion magnetic resonance imaging (dMRI) offer quantitative approaches to study skeletal muscle microstructure and architecture. Despite its prevalence in applications for musculoskeletal research, clinical adoption is hindered by a lack of understanding regarding its sensitivity to clinically important biomarkers such as muscle fiber cross‐sectional area. This review aims to elucidate how dMRI has been utilized to study skeletal muscle, covering fundamentals of muscle physiology, dMRI acquisition techniques, dMRI modeling, and applications where dMRI has been leveraged to noninvasively study skeletal muscle changes in response to disease, aging, injury, and human performance.

**Level of Evidence:**

5

**Technical Efficacy:**

Stage 2

Skeletal muscle is a vital organ system in the human body, playing a crucial role in metabolism, mobility, and postural stability.^1^ With disease and injury, micro‐ and macrostructural changes in skeletal muscle can occur, resulting in impaired function. Traditionally, muscle microstructure is evaluated via biopsy, which is invasive, destructive to the tissue, and provides information only about a relatively small volume of the overall tissue. Muscle macrostructure provides gross information about muscle (eg, volume and fat quantitation), but lacks sensitivity to cell or cell‐bundle level details that are important for function. This has driven interest in developing noninvasive techniques to evaluate muscle microstructure.

Diffusion magnetic resonance imaging (dMRI) is a noninvasive technique that has enhanced the ability of investigators to visualize and quantify tissue microstructure.^2^, ^3^ The utility of dMRI sequences enables comprehensive assessments of tissue integrity, composition, and function beyond what has been possible with conventional T1‐weighted or T2‐weighted musculoskeletal imaging techniques. This ability to noninvasively evaluate tissue microstructure facilitates a deeper understanding of structure–function relationships—unique features of skeletal muscle—providing a holistic view of skeletal muscle in the context of aging, injury, or disease.

dMRI encompasses a variety of techniques, all of which exploit the anisotropic movement of water molecules within restricted cellular environments.^4^ This technique enables quantitative analysis of microstructural changes in skeletal muscle that have traditionally been studied using animal models or biopsies of human tissue. The ability to noninvasively assess these biomarkers of tissue health facilitates the serial evaluation of a tissue over time, which allows for the effect of adjuvant therapeutics, disease processes, and the natural healing process to be evaluated. Furthermore, dMRI sequences are common across different MRI vendors, allowing for easy implementation of these protocols. In addition, a number of analysis tools are freely available, allowing for adoption of basic and complex dMRI techniques to be evaluated for a number of musculoskeletal injuries, diseases, and more. Despite its various capabilities and sensitivity in microstructural analysis, the application of dMRI in routine clinical practice remains limited. There is immense potential in the identification of novel diagnostic biomarkers that can predict pathological changes, evaluate therapeutic strategies, and guide surgical planning. The deployment of this technique in the clinic is currently limited by a lack of understanding on the sensitivity of dMRI to specific, clinically relevant biomarkers of muscle health.

Aside from microstructural assessments of muscle, dMRI can be used to model muscle macrostructure through the use of a postprocessing technique called tractography.^5^ This technique exploits the principle that diffusion magnitude is greatest in the direction along the main axis of a muscle fiber. Several algorithms have been developed to connect the primary axis of diffusion through all voxels within a dMRI experiment to generate estimations of gross muscle fiber length and pennation angle—key components of muscle architecture—and are referred to as fiber tracts. As muscle architecture measurements are traditionally performed on cadaver tissue, the ability to noninvasively assess muscle architecture in vivo has a number of potential applications, including for modeling joint behavior and helping to inform surgical decisions.

The overall goal of this review is to provide a baseline understanding of dMRI, how it can be leveraged to study skeletal muscle, and where this technique currently being employed. To provide a better understanding of what features of skeletal muscle are important from a muscle physiology perspective, a brief overview of key features of muscle microstructure and architecture in the context of whole tissue function is presented. Furthermore, fundamentals of routine and more complex dMRI data acquisition and analysis techniques is presented as well as context for how to improve the ability to understand the influence of tissue level changes on the measured diffusion signal. Finally, clinically relevant applications of these different dMRI techniques are presented, with a primary focus on publications from 2019 to 2024.

## A Brief Overview of Muscle Physiology

dMRI is a powerful tool that can be used to noninvasively probe both muscle microstructure and architecture. Muscle microstructure is directly related to the isometric force generating capacity of a muscle fiber.^6^ Thus, one of the core reasons to use dMRI to study skeletal muscle is to track changes in muscle fiber cross‐sectional area that are associated with muscle function. Commonly observed changes in muscle microstructure with injury or pathology include fiber atrophy/hypertrophy, fibrosis, membrane damage (permeable fibers), fatty infiltration, and edema, all of which can influence muscle function.

Skeletal muscle has a well‐characterized structure–function relationship, where the arrangement of muscle fibers can predict functional capacity. Skeletal muscle architecture is defined as “the arrangement of muscle fibers within a muscle relative to the axis of force generation.”^7^ While muscle fiber sizes are generally similar across muscles within the human body, architectural measurements are varied and are the best predictors of force generation.

The features of a muscle that are typically assessed when discussing muscle architecture are muscle mass (*M*_m_), sarcomere normalized fiber length (*L*_f_), and pennation angle (*θ*; fiber angle relative to the force‐generating axis). Together these parameters are used to calculate physiological cross‐sectional area (PCSA)—the sum of the cross‐sectional areas of all the muscle fibers within a muscle^8^—given the following equation:

(1) $$\text{PCSA}\;\left(mm^{2}\right)=\frac{M_{m}\left(g\right)\times \cos\left(\theta \right)}{\rho \left(\frac{g}{cm^{3}}\right)\times L_{f}\left(\mathrm{mm}\right)}$$

where *ρ* is muscle density (1.056 g/cm^3^ in humans). Given that muscle volumes (*V*_m_) can be computed from MRI images, *M*_m_/*ρ* can be replaced by *V*_m_. It is important to note fiber lengths are normalized to sarcomere length via the following equation:

(2) $$L_{f}=L_{f}^{\prime }\times \left(\frac{2.7\mathrm{\mu m}}{L_{s}^{\prime }}\right)$$

where $L_{f}^{\prime }$ is the gross measured muscle fiber length and $L_{s}^{\prime }$ is the measured sarcomere length. A length of 2.7 μm is the optimal sarcomere length for human muscle, where the sarcomere can produce the greatest isometric force according to the length‐tension curve. The maximum tetanic tension (*P*_0_) that a muscle can produce, based on its PCSA can be calculated by the equation^8^:

(3) $$P_{0}=\text{PCSA}\times \text{Specific tension}$$

where the specific tension of muscle is assumed to be 22.5 N/cm^2^.^9^, ^10^

Taken together, muscle microstructure and muscle architecture are key features of skeletal muscle related to muscle function. However, while muscle $L_{f}^{\prime }$ and *θ* can be obtained from dMRI‐based tractography, $L_{f}$ requires obtaining sarcomere length measurements, which to date can only be performed using invasive laser diffraction or polarized light microscopy techniques. Therefore, true PCSA—although often incorrectly reported in dMRI studies—cannot be made using tractography alone. Albeit, the ability to noninvasively assess muscle microstructure and architecture in vivo provides a powerful tool to better understand tissue level adaptations to various stimuli in order to predict clinically meaningful functional outcome measures.

## dMRI Techniques for Skeletal Muscle Imaging

### Microstructural Influence on Water Diffusion in Skeletal Muscle

**Figure 1:**
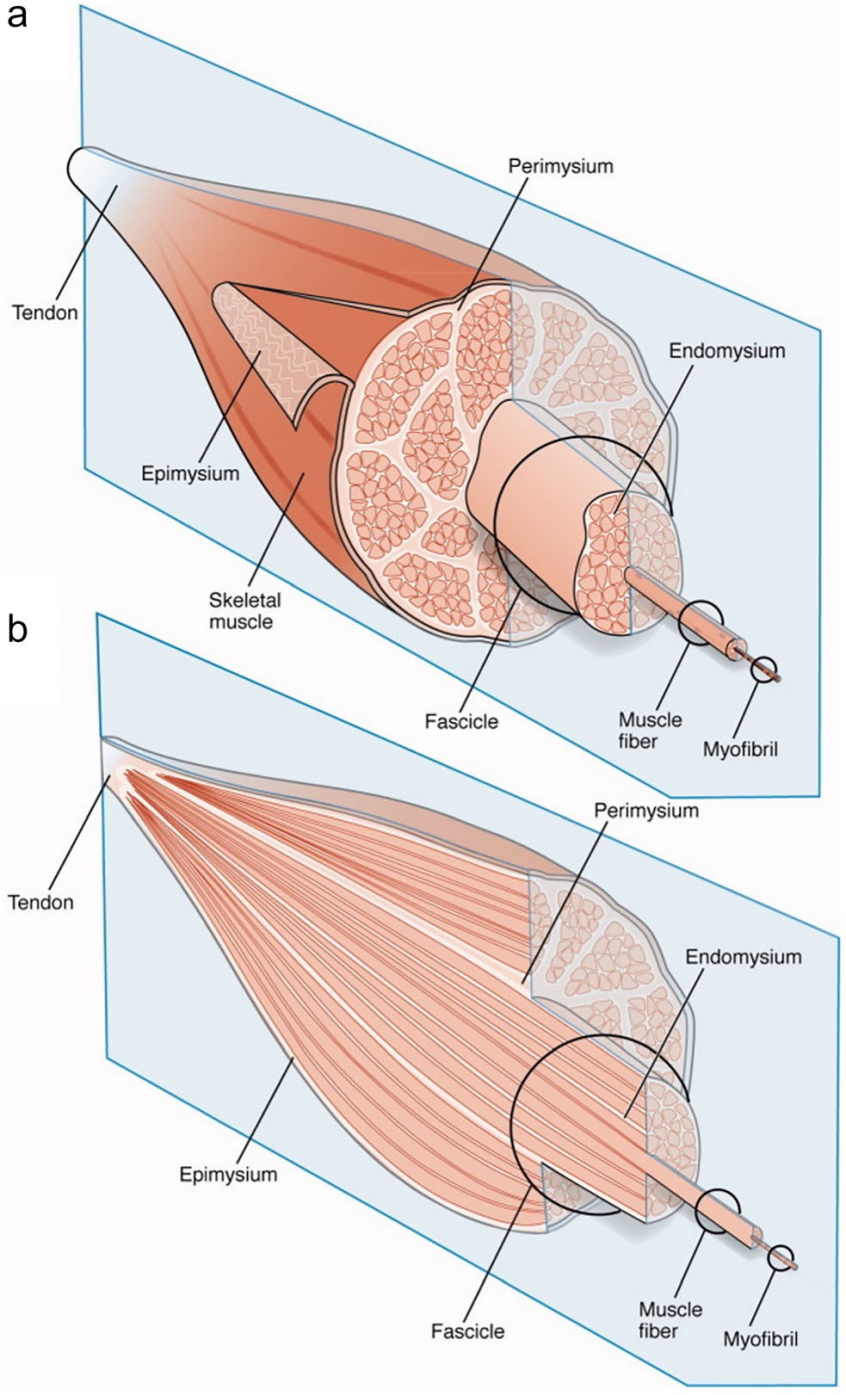
Schematic diagram of the gross organization of unipennate muscle tissue and muscle extracellular matrix–tendon organization. (**a**) Muscle has a hierarchical structure, sarcomeres—the fundamental contractile unit of muscle contraction—are organized into myofibrils, which are further bundled together to form muscle fibers. Muscle fibers are elongated cells comprising of numerous myofibrils and are enclosed in a sarcolemma (cell membrane). Muscle fibers are bundled together by connective tissue called the endomysium, into larger structures called fascicles, which are surrounded by an additional layer of connective tissue called the perimysium. A whole muscle is a collection of fascicles organized into a specific architecture to support the unique function of a specific muscle. (**b**) Cross‐section of a muscle demonstrating anisotropic muscle fibers. Figure reproduced with permission Wiley Periodicals (2011).^**11**^

Water molecules in biological tissues exhibit random, thermally linked motion called Brownian motion. Diffusion of these molecules is influenced by local microstructural features of the tissues in which they are diffusion including cell membranes. Water molecules in skeletal muscle exhibit anisotropic diffusion, meaning that water molecules will preferentially diffuse along the main axis of a muscle fiber compared to radially across a muscle fiber, due to its inherent microstructural properties.^4^ Skeletal muscle has a highly organized microstructure that is directly related to whole muscle function^1^ (Fig. 1). The fundamental contractile unit of muscle contraction is the sarcomere, which is comprised of the contractile proteins actin and myosin. Sarcomeres are organized into myofibrils, which are further bundled together to form muscle fibers (cells). In humans, these cells are 40–70 μm in diameter. Muscle fibers are elongated cells comprised of numerous myofibrils and are enclosed in a sarcolemma (cell membrane). Muscle fibers are bundled together by connective tissue called the endomysium, into larger structures called fascicles, which are surrounded by an additional layer of connective tissue called the perimysium. In humans, fascicles are on the millimeter size scale, which makes them the size‐relevant structure for dMRI resolutions. A whole muscle is a collection of fascicles organized into a specific architecture to support the unique function of a specific muscle.

### dMRI Acquisition Sequences and Protocols

Many variants of dMRI pulse sequences have been implemented to assess microstructural features of skeletal muscle at different anatomical scales. Each sequence offers specific advantages and limitations in imaging skeletal muscle. The choice of dMRI pulse sequence depends on the specific research or clinical goals of the study and sensitivity to specific microstructural features. However, there are trade‐offs in acquisition times, technical complexity, and susceptibility to artifacts with each of these sequences that must be additionally taken into account. A brief summary of the below dMRI pulse sequences and protocols can be found in Table 1.

**Table 1:** Different Pulse Sequences Available for dMRI Studies of Skeletal Muscle

Pulse Sequence	Availability	Strengths	Weaknesses
Spin echo sPFG	Most common	Widely used	Limited sensitivity
		High SNR	Long echo times can lead to decreased SNR
		Tractography	
Stimulated echo sPFG	Not common, can be requested from vendor	Long effective Δ without T2 decay	Low SNR
		Increased sensitivity to fiber size	Difficult to implement
dPFG	Uncommon, requires pulse sequence programming	High sensitivity to microscopic anisotropy	Highly sensitive to motion and timing errors
		Provides detail on compartment size and shape	Requires complex data analysis/does not fit the standard diffusion tensor model
			Lower SNR than sPFG
OGSE	Not common, can be requested from vendor	High temporal resolution	Difficult to implement
		High SNR	Higher sensitivity to hardware limitations
		Sensitive to SMAMs	

sPFG = single‐pulsed field gradient; dPFG = double‐pulsed field gradient; OGSE = oscillating‐gradient spin‐echo; Δ = diffusion time; SNR = signal‐to‐noise ratio; SMAMs = spontaneous mechanical activities of musculature.

One of the key terms that is used in dMRI is the *b*‐value, which is the amount of diffusion weighting that is applied during an experiment to collect a diffusion‐weighted image (DWI). The definition of *b*‐value for a trapezoidal (most commonly utilized) dMRI gradient is:

(4) $$b=\gamma^{2}G^{2}\delta^{2}\left(\Delta -\frac{\delta }{3}\right)$$

where *γ* is the gyromagnetic ratio, *G* is the gradient amplitude, *δ* is the time a gradient is applied, and Δ is the duration between paired gradients. For skeletal muscle, *b*‐values between 400 and 500 seconds/mm^2^ are typically used.^12^ Note, for nontrapezoidal gradients, the *b*‐value can be calculated using the following equation:

(5) $$b=\gamma^{2}\int_{0}^{\Delta }{\left(\int_{0}^{t}G\left(t^{\prime }\right)dt^{\prime }\right)}^{2}\mathrm{dt}$$

where *G*(*t*) is the time‐dependent gradient strength.

**Figure 2:**
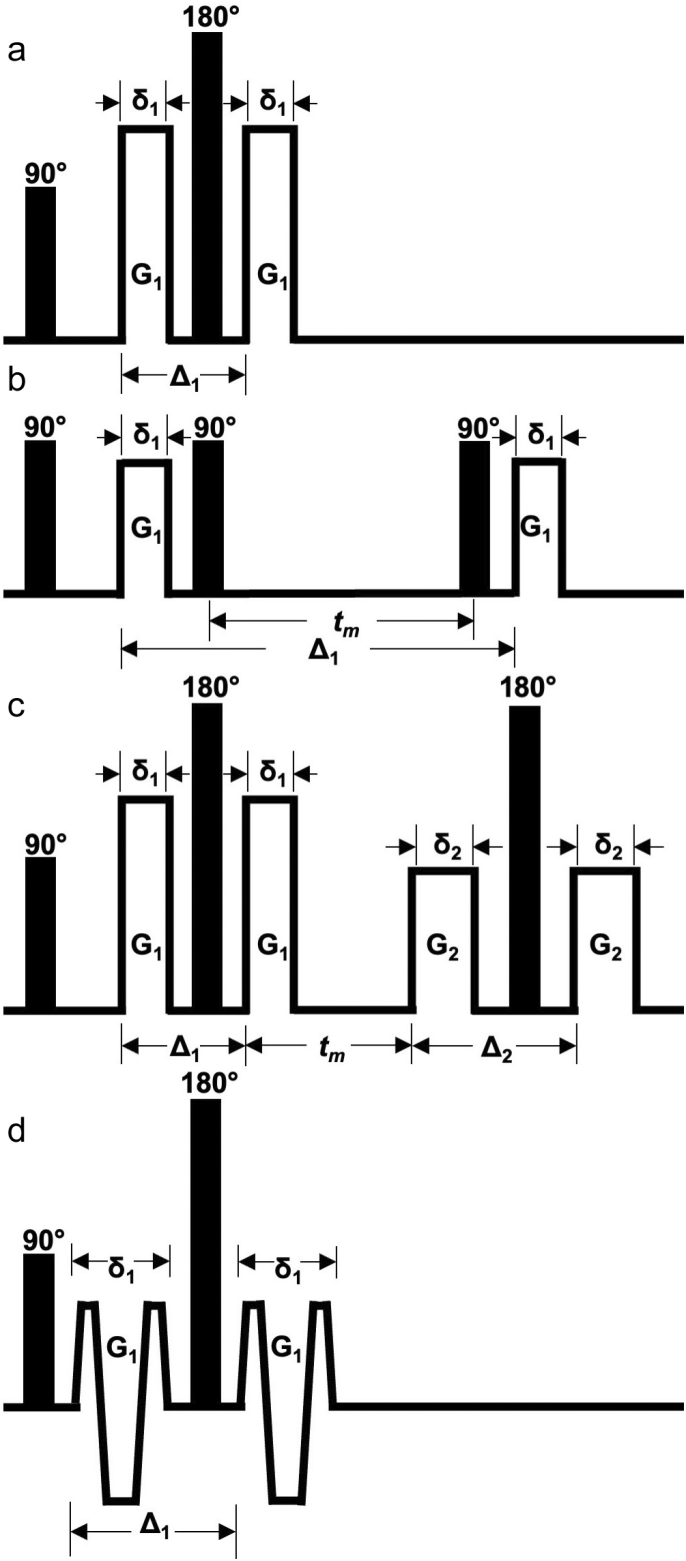
Pulse sequence diagrams of (**a**) spin echo single‐pulsed field gradient, (**b**) stimulated echo single‐pulsed field gradient, (**c**) double‐pulsed field gradient, and (**d**) oscillating‐gradient spin‐echo dMRI. Gradient strength (*G*), duration (*δ*), and diffusion time (Δ) are listed for each pulse sequence. Radiofrequency pulses are represented by black bars.

The workhorse of dMRI pulse sequences is the single‐pulsed field gradient (sPFG) spin echo sequence, where a 90° radiofrequency (RF) pulse is applied, followed by a set of diffusion‐encoding gradients around a 180° refocusing pulse (Fig. 2a). This sequence is provided by most vendors, and is most commonly used to study muscle microstructure. These sequences are best applied when trying to achieve higher resolution scans, or for tractography, as they have relatively high signal‐to‐noise ratio (SNR) in comparison to other dMRI methods. Main limitations of this sequence include relatively short Δ that can be probed (on the order of 15–30 msec) due to the need for short echo time (TE) when imaging muscle, which has a relatively short T2; ~29 msec at 3.0 T and ~23 msec at 7.0 T.^13^, ^14^

Stimulated echo acquisition mode (STEAM) sPFG dMRI sequences can be employed to probe long Δ, increasing sensitivity to muscle fiber size. However, there are trade‐offs in SNR—stimulated echo dMRI has SNR half that of spin echo given the same imaging parameters—that necessitate the use of larger voxel sizes, decreasing spatial resolution. These sequences employ a 90° RF pulse, followed by a diffusion gradient, followed by another 90° RF pulse to tip dephased spins into the longitudinal (along the bore of the magnet), preventing T2 decay until a third 90° RF pulse flips the spins into the transverse plane where an additional diffusion gradient is applied (Fig. 2b). By increasing the mixing time (time between the second and third RF pulse), the Δ is increased. When performing these scans, there is a contribution of the imaging gradients to the overall diffusion signal resulting in increased effective *b*‐values and directional weighting along the longitudinal plane.

**Figure 3:**
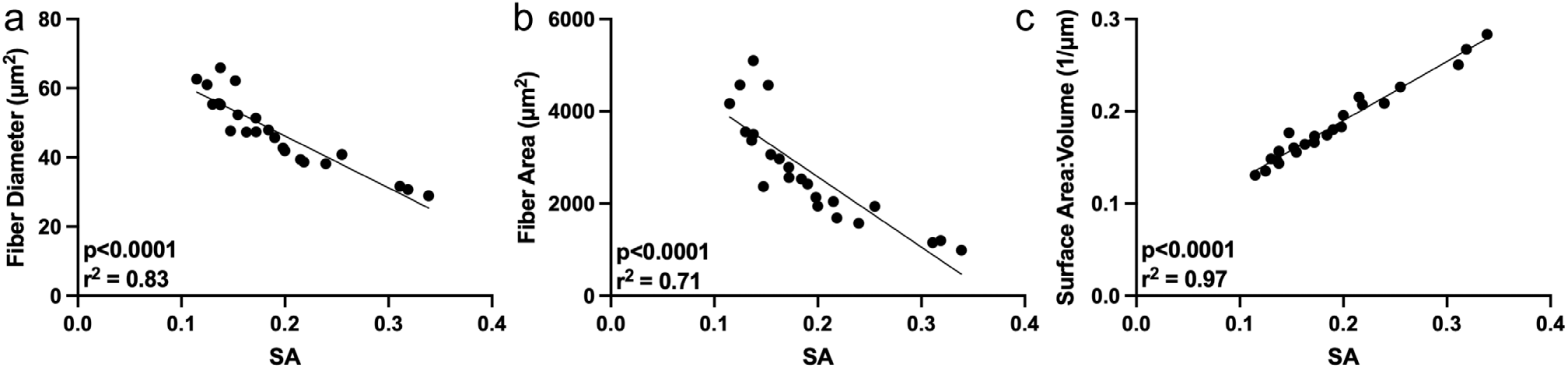
Correlations between spherical anisotropy (SA) and fiber diameter (**a**), fiber area (**b**), and surface area to volume ratio (**c**) from simulated models of muscle microstructure. SA is a scalar measurement of double‐pulsed field gradient dMRI data that is calculated from diffusion tensor subspace imaging (DiTSI). SA describes the angular variance of the diffusion signal and varies from 0 to 1 and can be thought as analogous to FA. Figure reproduced with permission Wiley Periodicals (2023).^17^

In general sPFG sequences are similar as they measure the diffusion‐weighted signal along a specified set of angular directions, providing valuable information about average microstructure (i.e., macroscopic anisotropy) but not the variance in microstructure within a voxel (i.e., microscopic anisotropy), which is a key biomarker of some forms of muscle pathology.^6^, ^15^ This is a fundamental limitation of sPFG acquisitions. Overcoming this limitation requires an acquisition with multiple diffusion encodings in a single echo time, called multivector methods,^16^ the simplest of which is double PFG (dPFG) (Fig. 2c). This sequence is similar to a spin echo sPFG sequence, except that a second pair of diffusion encoding gradients is applied after the first, resulting in complex pathways of diffusion being measured. While the potential advantages of these methods are known, their utility has not been fully exploited because of a lack of sequence availability and a robust analysis method capable of fully utilizing the complex information they provide. A recent study has demonstrated that a dPFG dMRI pulse sequence plus analysis may result in increased sensitivity to muscle fiber microstructure than the sPFG‐based DT model (Fig. 3); however, further studies are needed to validate this approach.^17^

Oscillating gradient spin echo (OGSE) pulse sequences are inherently different from PFG sequences as they replace the pulsed gradients with oscillating gradients (Fig. 2d). This technique is interesting as Δ is not dependent on the separation of the gradients, but is determined by the oscillating period. This allows for very short Δ to be probed, making this approach favorable for studying microstructure in actively contracted muscles.

### Quantitative Models of Restricted Diffusion

The most common model used to quantify dMRI data is the diffusion tensor (DT), a second‐order tensor that quantifies the magnitude and direction of bulk diffusion within a given voxel. To calculate the DT, a minimum of six diffusion encoding directions must be measured, with more diffusion directions resulting in higher angular resolution and better overall estimates of the DT. The tensor can be diagonalized using an eigenvector and eigenvalue decomposition, resulting in three orthogonal eigenvectors, that point along the principle (longitudinal) axis of diffusion (*ε*_1_) and radial axis of diffusion (*ε*_2_, *ε*_3_). The corresponding eigenvalues quantify diffusion along these eigenvectors, and *λ*_1_ ≥ *λ*_2_ ≥ *λ*_3_. As *λ*_2_ and *λ*_3_ are oriented orthogonal to the long axis of a muscle fiber, these parameters are thought to be most sensitive to muscle fiber size. Several scalar‐based indices of the DT are commonly reported in order to quantitatively compare diffusion between muscles, subjects, scanners, and serially over time. Mean diffusivity (MD) quantifies the overall diffusion within a voxel:

(6) $$\mathrm{MD}=\bar{\lambda }=\frac{\lambda_{1}+\lambda_{2}+\lambda_{3}}{3}$$

Radial diffusivity (RD) is the average of the radial components of the DT:

(7) $$\mathrm{RD}=\frac{\lambda_{2}+\lambda_{3}}{2}$$

Fractional anisotropy (FA) is unitless and describes the shape of the DT from 0 (perfectly isotropic) to 1 (perfectly anisotropic):

(8) $$\mathrm{FA}=\sqrt{\frac{3}{2}}\frac{\sqrt{{\left(\lambda_{1}-\bar{\lambda }\right)}^{2}+{\left(\lambda_{2}-\bar{\lambda }\right)}^{2}+{\left(\lambda_{3}-\bar{\lambda }\right)}^{2}}}{\sqrt{\lambda_{1}^{2}+\lambda_{2}^{2}+\lambda_{3}^{2}}}$$

**Figure 4:**
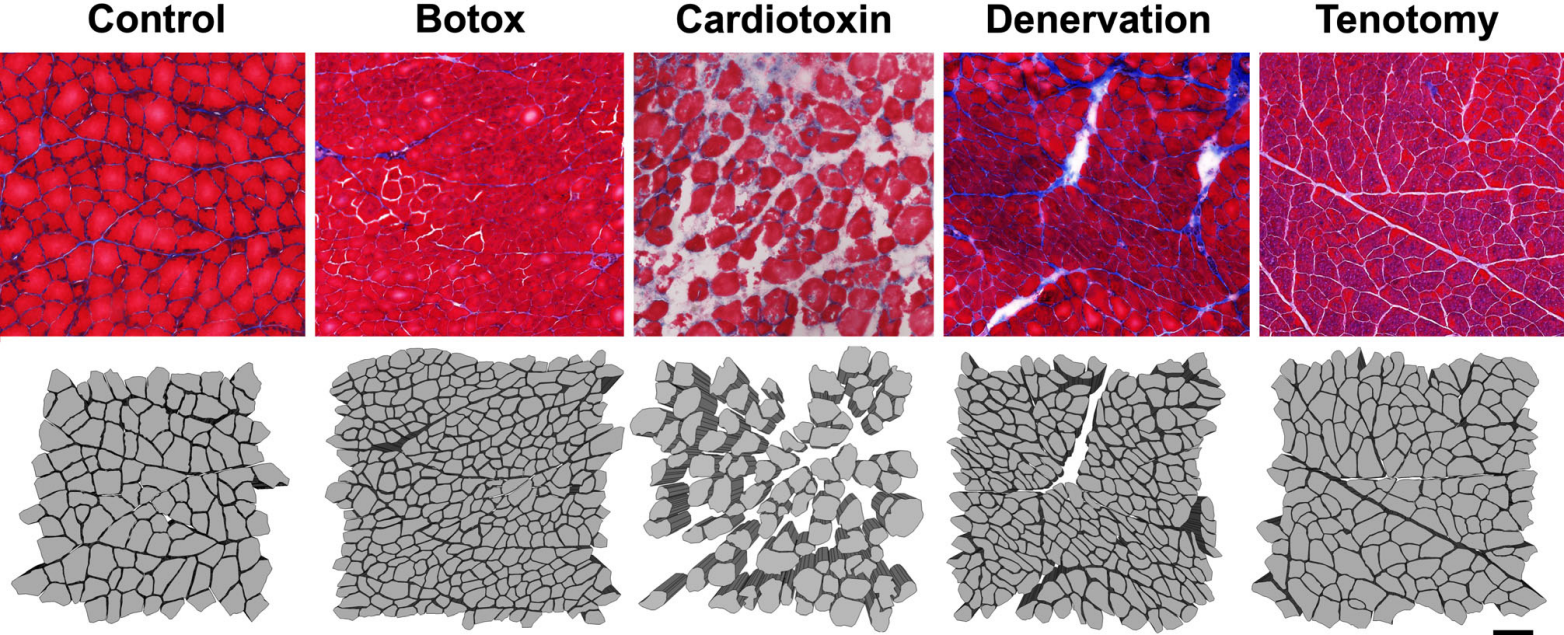
Schematic depicting histology informed models of skeletal muscle. Botox, denervation, and tenotomy models are from animals 30 days postinjury. Cardiotoxin models are from animals 3 days postinjury. Scale bar indicates 100 μm. Figure reproduced with permission from Wiley Periodicals (2021).^18^

Generally, as a muscle fiber cross‐sectional area increases, *λ*_2_ and *λ*_3_ increases, resulting in increased RD and decreased FA and vice versa. However, these relationships become more complex when the muscle is inflamed, has damage to the sarcolemma, fatty degeneration is present, or becomes fibrotic. Examples of histology and geometric models generated from complex muscle injury models in animals are presented in Fig. 4. These geometric models have been employed to explore relationships between tissue microstructure and dMRI measures using spin‐echo sPFG,^19^ stimulated echo sPFG,^18^ and dPFG pulse sequences.^17^

The DT is the most commonly utilized way to analyze dMRI data. However, several other methods have been developed to interpret and quantify dMRI data. Multicompartment models have been applied to separate diffusion of water species based on native T2 relaxation. For example, Fan and Does utilized a multiecho spin echo sPFG pulse sequence to simultaneously measure restricted diffusion and T2 decay in normal and inflamed skeletal muscle.^20^ By fitting the data to a two‐compartment model, the restricted diffusion of the intracellular and extracellular components were able to be independently measured. This application has potential to enhance sensitivity to microstructural changes that occur in inflamed muscle.

To date, the most commonly employed approach to model the microstructural features that inform the diffusion signal is the random permeable barrier model^21^ (RPBM), which describes diffusion of water molecules in permeable, randomly oriented membranes. Briefly, the input arguments to the model are RD(Δ) and $\lambda_{1,\Delta >100\text{msec}}$. From this model the free diffusion coefficient (*D*_0_), the characteristic time scale associated with a single membrane (*τ*), and the effective “volume fraction” (*ζ*) are fit using nonlinear least squares analysis. From these parameters, features of muscle microstructure (surface area to volume [S/V] ratio and average fiber size [*a*] can be derived, as well as an estimate of the permeability (*ε*) of the sarcolemma.^22^ Generally, the microstructural effects observed with RPBM reflect expected changes in the microstructure that result from: increasing fiber size during postnatal growth,^23^ postexercise fiber dilation in healthy patients but not in patients with chronic extracellular compartment syndrome,^24^ decrease in muscle fiber size with age,^23^ disuse atrophy and recovery,^25^ and decreased fiber size after rotator cuff repair.^22^ This technique has been shown to accurately estimate S/V from in vivo animal^23^ and in silico studies.^18^ RPBM is the closest model we have to predict fiber size noninvasively and it has been explored by several groups. However, it is important to note that interpreting the findings as absolute measurements of muscle fiber size may not be exact. In independent studies that have patient‐matched biopsies, no agreement has been found between RPBM predicted fiber size and histologically measured fiber size,^26^, ^27^ though it is difficult to precisely collocate and interpret biopsy measurements relative to whole muscle measurements.

Forward‐simulated model is a technique in which a dictionary‐based approach is used to predict metrics of muscle microstructure given measured dMRI data. Generally, these approaches have not yet been validated against histology, and when applied in real experiments, predict fiber sizes larger than what has been previously reported in the literature.^28^, ^29^ However, the microstructural effects observed reflect expected changes in microstructure (i.e., smaller muscle fiber sizes associated with denervation).

dMRI data collected with dPFG pulse sequences do not fit the standard DT model. While there is currently no standard model to fit dPFG data, recent preliminary work has demonstrated that the diffusion tensor subspace imaging (DiTSI) model^30^ has high sensitivity to measuring both muscle fiber size and S/V and is postulated to have sensitivity to microscopic anisotropy within a voxel.^17^

Intravoxel incoherent motion (IVIM) MRI is a dMRI‐based technique that is sensitive to the intravascular motion of blood flowing in randomly oriented capillaries in a tissue as well as the molecular diffusion of water in the intracellular and extracellular space.^31^ IVIM utilizes diffusion‐weighted MRIs over a wide range of *b*‐values, typically 20–700 seconds/mm^2^. The scale of the motion of the intravascular and extravascular spaces differs by an order of magnitude, and the intravascular component of the MRI signal is not detectable at *b*‐values greater than 200 seconds/mm^2^ due to the attenuated signal from rapidly diffusion water in this space. Thus, the measured data can be fit to a biexponential model that describes the contribution of signal from the intravascular (referred to as the pseudo‐diffusion coefficient, D*), and extravascular (molecular diffusion coefficient, *D*) spaces, weighted by the intravascular signal fraction (*f*) to signal decay as a function of *b*‐value:

(9) $$\frac{S\left(b\right)}{S_{0}}=\left(1-f\right)e^{-\mathrm{bD}}+fe^{-bD^{*}}$$

Depending on how diffusion data are acquired, it is possible to combine volumes used to calculate both IVIM and the DT from a single pulse sequence. Furthermore, there are many approaches to fit the resulting diffusion data to the IVIM equation; however, a recent analysis by Englund et al demonstrated that a three‐step nonlinear least squares fitting approach, where *D*, *f*, and D* were estimated sequentially, generally yielded the lowest spatial and temporal variations.^32^ For a detailed recent review on IVIM in skeletal muscle, refer to Englund et al.^33^

Diffusion Kurtosis imaging (DKI) is a dMRI technique that extends the capabilities of DTI by characterizing non‐Gaussian diffusion behavior in biological tissues.^34^ It quantifies the degree of tissue complexity and heterogeneity by measuring the diffusion kurtosis, providing additional insights beyond traditional diffusion metrics. DKI requires dMRI data collected at a number of *b*‐values to fit to the following equation:

(10) $$\ln\left(\frac{S_{b}}{S_{0}}\right)=\mathrm{bD}+\frac{1}{6}b^{2}D^{2}K$$

where *S*_*b*_ represents the signal intensity at a specific *b*‐value, *S*_0_ is the signal intensity at *b* = 0 seconds/mm^2^, *D* represents the diffusion coefficient, and *K* represents kurtosis.

### Spontaneous Mechanical Activities of Musculature

Achieving an adequate SNR is essential for DWI of skeletal muscle, and subsequent calculation of the DT. A minimum SNR of 25 is recommended for calculating the DT,^12^ with higher SNR resulting in more accurate calculations.^35^ Selecting the appropriate parameters to achieve both adequate SNR and effective diffusion of water in skeletal muscle presents several challenges.^12^, ^35^ There is an inverse relationship between diffusion magnitude and SNR, such that a greater *b*‐value leads to a lower SNR in DT‐MRI. However, the main limitation in SNR comes from the increased TE necessary for higher *b*‐values; it is technically challenging to quickly turn on and off gradients with sufficient gradient strength to achieve higher *b*‐values while maintaining a short TE. Long TEs result in reduced baseline SNR due to the relatively short T2 of muscle as mentioned earlier.

The DT describes the directionality of water diffusion in human tissues and is a crucial step in estimating muscle architecture via tractography.^3^, ^36^ Precautions are generally taken to limit bulk motion caused by participant discomfort or scanner vibration. However, SNR is also known to be susceptible to variations in field strength, tissue composition, and partial volume effects, all of which pose obstacles to collecting valid and reliable data. Early muscle diffusion studies also observed spontaneous signal dropout, referring to localized areas of significantly reduced signal intensity or signal voids.^37^, ^38^, ^39^, ^40^ The primary cause of these signal voids has been attributed to sporadic motor unit activity, referred to as spontaneous mechanical activities of musculature (SMAMs), that is, a muscle twitch. This localized incoherent motion leads to additional decrease in SNR, significantly impacting the ability to accurately quantify the diffusive (eg, MD, FA) and architectural (eg, fascicle length, pennation angle) properties of skeletal muscle noninvasively.

**Figure 5:**
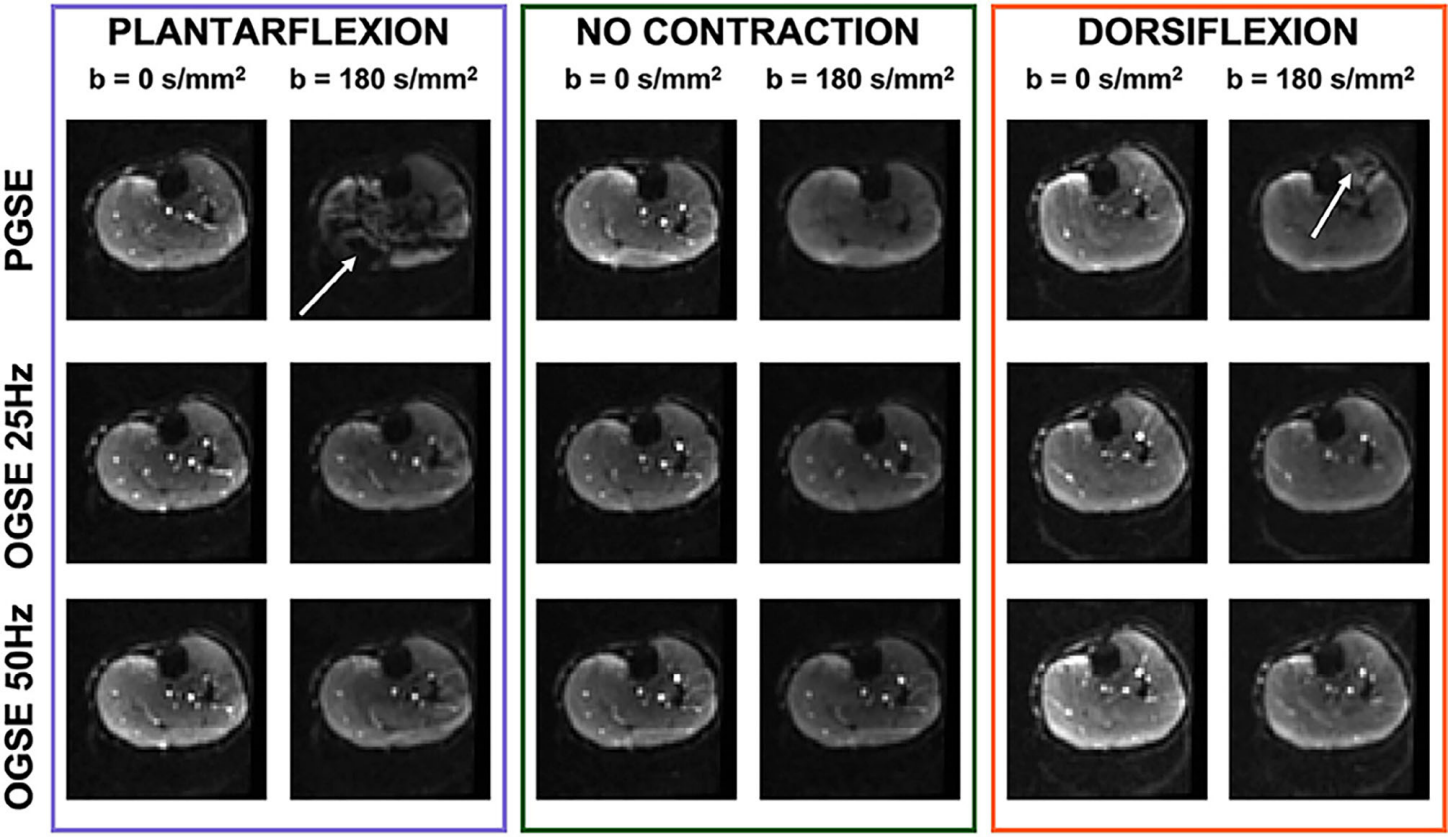
Nondiffusion‐weighted and diffusion‐weighted dMRI of a volunteer acquired during active plantarflexion (purple), with relaxed musculature (no contraction, green), and during active dorsiflexion (orange). Data acquired from single‐pulsed gradient spin echo (PGSE) dMRI sequences show localized areas of signal voids (white arrow) originating by spin dephasing during muscle activation that is not observed during oscillating gradient spin echo (OGSE) dMRI sequences. Figure reproduced with permission from Frontiers (2021).^44^

Several studies have sought to elucidate the causes of signal dropout and the effects of signal voids on diffusion analysis.^41^, ^42^, ^43^, ^44^ These studies have concluded that SMAMs are the primary cause of these signal voids, noting greater signal homogeneity in relaxed musculature compared to contracted muscles (Fig. 5). This hypothesis is supported by previous work comparing patients with amyotrophic lateral sclerosis (ALS) to healthy controls, observing more muscle twitches and signal voids in the ALS patients.^45^ The current literature suggests that the appearance of these signal voids does not follow a consistent pattern, but can be influenced by factors such as pulse sequence or level of compaction (i.e., spacing of motor unit territories).^43^, ^44^, ^46^ Furthermore, SMAMs are more pronounced in stimulated echo dMRI sequences as they allow more time for motion during Δ.^47^ A study by Mazzoli et al reported an overestimation of diffusion parameters and up to a 500% increase in signal voids in the calf with a pulsed gradient spin echo sequence compared to OGSE sequences.^44^ Although compelling data exist to help us understand the cause of signal dropout, the effects of signal voids on data processing and analysis have not been fully clarified.

Future studies should consider strategies to mitigate the occurrence of SMAM‐induced signal dropout, improving image quality, diagnostic accuracy, and quantitative analysis. These advancements are critical to increase the efficacy of musculoskeletal models, and enhance the precision of clinical assessments or procedures (eg, surgery). Determining the effects of different *b*‐values, gradient orientations, and vendors have been explored in attempts to reduce the occurrence and magnitude of signal voids.^46^, ^48^ This work can be advanced by evaluating differences among various MR coils, muscle groups, and field strengths to determine if these factors have a significant effect on the number or intensity of signal voids. Acquisition parameters in sequence development are also important to consider, as quick rise times of gradient coils can induce peripheral nerve stimulation, potentially causing spontaneous muscle activity in single fibers or localized regions.^49^, ^50^ Continued development and optimization of pulse sequences remain one of the best solutions to increase uniform SNR in skeletal muscle MRI.^44^, ^46^, ^51^ Additionally, standardizing some of the steps for postprocessing and analysis may be useful in mitigating the extent and frequency of the signal voids caused by SMAMs. Some postprocessing algorithms have already been proposed.^37^, ^52^

### Fatty Infiltration

Fatty infiltration—also known as fatty degeneration—refers to a pathophysiologic process resulting in the abnormal accumulation of fat within and around muscle tissue. These changes can be in addition to existing lean tissue and/or in place of pathologic muscle tissue. Common in diseases such as muscular dystrophies, polymyositis, and with normal aging, there is a progressive increase in the total amount of fat located within the fascial planes of a muscle belly, which can complicate assessment of muscle health with dMRI techniques. Fat has a longer T2 relaxation and a decreased overall diffusivity compared to water in skeletal muscle, thus the inclusion of fat in dMRI measurements can lead to incorrect interpretation of underlying microstructure.

To suppress the effect of fat on dMRI in skeletal muscle, several techniques have been employed. The most common way to attenuate the effect of fat is spectral attenuated inversion recovery (SPAIR), where an adiabatic inversion pulse tuned to the resonance of fat inverts the longitudinal magnetization of fat to the −*z* direction. When the longitudinal magnetization of fat is zero, the water proton imaging sequence is initiated. SPAIR is effective at robust suppression of the fat signal originating from methylene (—CH2) and methyl (—CH3) peaks, but does not suppress the olefinic peak (~5%–10% of signal) due to its resonance frequency close to water. Several approaches to enhance fat suppression during dMRI experiments have been developed, including spectral olefinic fat suppression (SOFS) and Dixon olefinic fat suppression (DOFS). In SOFS, the olefinic fat peak is suppressed with a second spectral saturation pulse centered at the olefinic resonance.^53^ In DOFS, a magnitude‐based chemical shift species approach is used based on prior information from *b* = 0 acquisitions.^54^ Careful application of fat suppression techniques should be implemented in dMRI studies of skeletal muscle. These techniques improve the accuracy, reliability, and interpretation of dMRI measurements, ensuring that the resulting quantitative measurements of restricted diffusion more accurately reflect true microstructural features of skeletal muscle tissue.

## Characterization of Skeletal Muscle Architecture Using DT‐MRI

**Figure 6:**
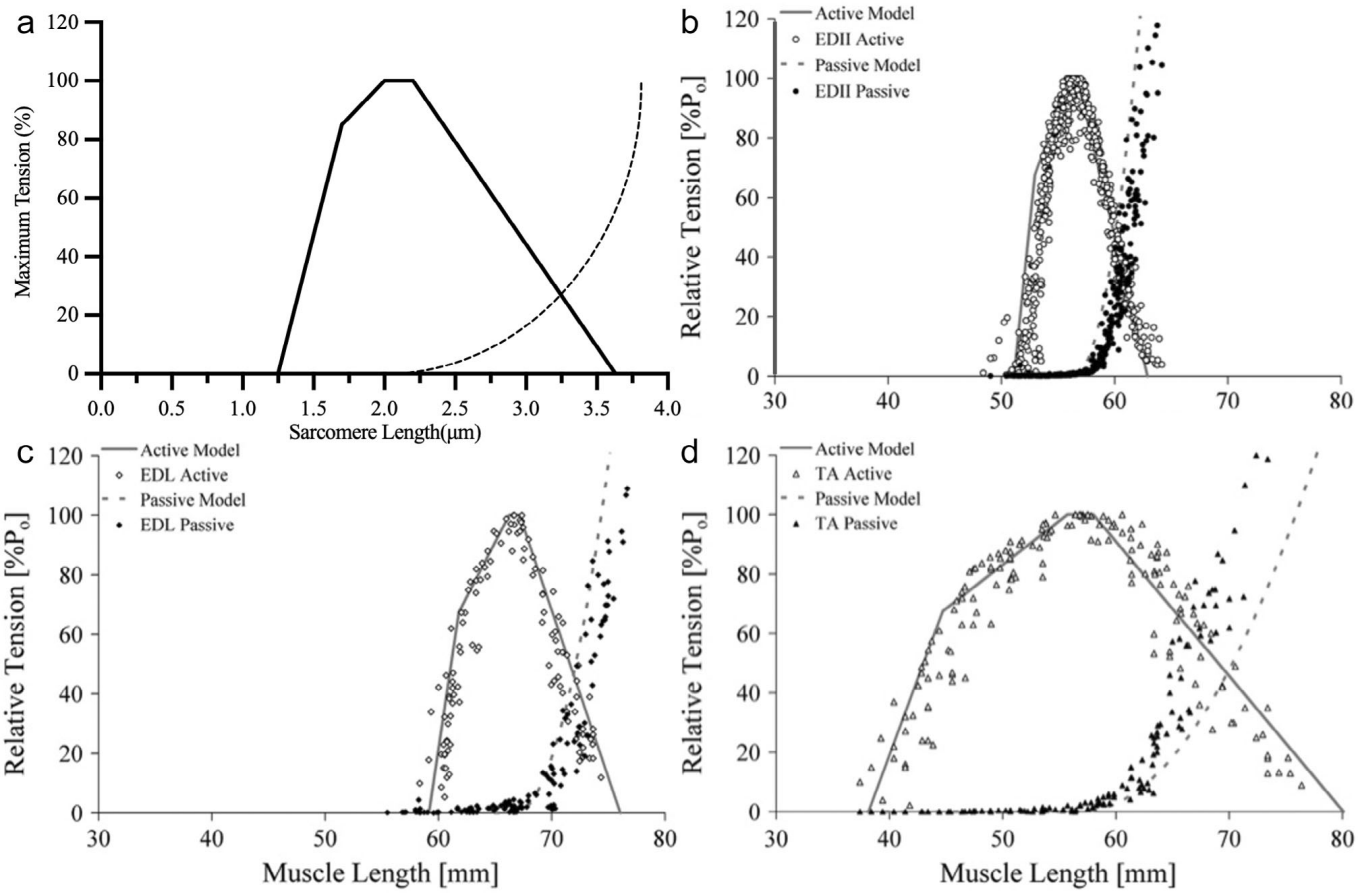
(**a**) Sarcomere length operating range and tension of human skeletal muscle. Active tension (solid line) and passive tension (dashed line) are both graphed. (**b**–**d**) Relationship between active theoretical (solid gray line) and experimental (open symbols) whole muscle length‐tension relationship and passive theoretical (dotted gray line) and experimental (filled symbols) whole muscle length‐tension relationship for rabbit. (b) Extensor digitorum II, (c) extensor digitorum longus, and (d) tibialis anterior muscles. This demonstrates that the fundamental length‐tension relationship that exists at a sarcomere scale extends to the muscle fiber scale. Thus, when measuring muscle fiber length for physiological cross‐sectional area measurements, it is necessary to understand sarcomere length in order to predict muscle tension. Figure reproduced with permission from Elsevier (2011).^55^

Key components of muscle architecture can be noninvasively estimated through an imaging technique called tractography, which is a postprocessing algorithm applied to dMRI data. This technique leverages the orientation of the principle eigenvector—which should be aligned with the long axis of a muscle fiber—to approximate tracts through an imaging volume. From tractography, it is possible to approximate muscle fiber length and orientations, which are key input parameters to approximate a muscle PCSA, and thus understand its function. While approximations of PCSA can be calculated, these measurements should not be considered as equivalent or superior to dissection or architecture‐based measured PCSA of a skeletal muscle due to the lack of sarcomere length information. The maximum tension that a muscle fiber can produce is directly related to underlying sarcomere length (Fig. 6a). Depending on muscle architecture, the sarcomere length‐tension curve scales to the whole muscle length tension curve (Fig. 6b–d).^55^ Therefore, reports of PCSA without underlying sarcomere length information are unable to accurately assess and model the force‐generating capacity of a muscle.

**Figure 7:**
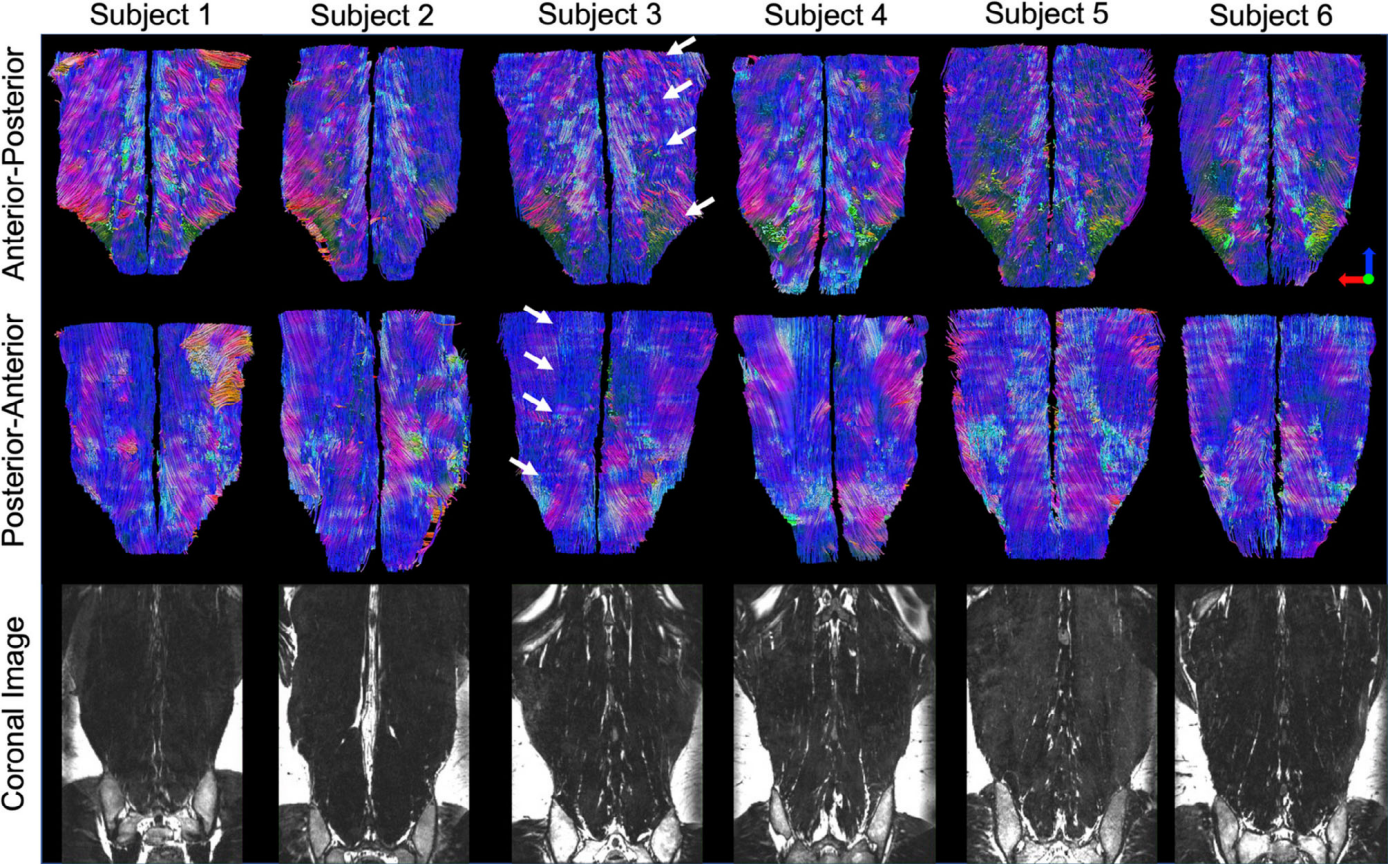
Representative tractography images of the lumbar spine from six active‐duty Marines (top and middle rows) with corresponding high‐resolution reformatted coronal anatomical MRIs (bottom row). Colors in the tractography scans indicate direction of the tracked fibers (blue—superior/inferior; red—medial/lateral; green—anterior/posterior). These images demonstrate subject‐to‐subject variation in lumbar muscle size and fiber orientation. The border between the iliocostalis and longissimus muscles is highlighted by white arrows for one subject, which cannot be seen in the corresponding anatomical image. Figure reproduced with permission from Wiley Periodicals (2020).^58^

Tractography does have several advantages over traditional, dissection‐based approaches to measuring muscle architecture, including: 1) the ability to measure fiber length and pennation angle in living organisms; 2) investigating the distribution of fiber lengths throughout the entire volume of a muscle^56^; 3) assessment of fibers deep within a muscle; 4) description of the three‐dimensional (3D) pennation angles, which has clear benefits for joint modeling; 5) assessment of fiber tracts in muscles undergoing fatty degeneration^57^; and 6) investigation of the heterogeneity of fiber lengths and pennation angles across subjects^58^ (Fig. 7).

Several groups have utilized tractography to approximate muscle fiber length and orientation (i.e., pennation angle) in a number of muscles, including pelvic floor,^59^ leg,^60^, ^61^ back,^58^, ^62^ and shoulder.^60^, ^61^ These outcomes have been evaluated in adult, adolescent,^63^ and infant^64^ populations. Furthermore, groups have investigated the role of diffusion acquisition parameters, SNR, and tractography algorithms for estimating muscle fiber length and pennation angle with tractography.^12^, ^65^

While widely used, there remains a lack of studies exploring the accuracy of tractography‐based architecture measurements with the tissue that is physically being scanned, with most studies comparing tractography measurements to previously reported architecture measurements in different cohorts. Three studies have directly compared tractography to measured architecture in the same tissue.^36^, ^66^, ^67^ Damon et al demonstrated the ability to resolve pennation angle in the tibialis anterior muscle of five mouse legs (*r* = 0.89).^36^ Schenk et al evaluated both fiber length and pennation angle in a single rabbit leg, and found no difference between dissection‐ and tractography‐based fascicle lengths but a mean difference of a 1.2° underestimation of pennation angle using tractography‐based assessment, which is still highly accurate.^66^ While this technique is widely used, there is still a need to validate tractography‐derived parameters in paired imaging‐ and dissection‐based studies in the same tissue in larger and more complex muscles than those in mouse or rabbit. In a study by Charles et al, direct relationships between lower‐limb muscles in fresh‐frozen cadaver tissue were measured with DTI and dissection‐based architecture measurements and found <5% differences in the two measurement techniques.^67^ However, freezing does create tissue microstructure damage, and thus may have an impact on tractography quality. While cadaver has traditionally been viewed as the best tissue to study, there are often image acquisition hurdles associated with crosslinked, dehydrated, or room temperature tissue.

It is worth noting that fiber tracts are not to be confused with actual muscle fibers, as the scale of a muscle fiber (tens of microns), is often much smaller than a traditional voxel size (millimeter scale). Thus, there are potentially thousands of muscle fibers that are passing through a voxel at a given location.

## DT Indices in Relation to Skeletal Muscle Function

Human performance is a key area of interest where dMRI has been leveraged to assess microstructural changes associated with muscle function. As skeletal muscle function is directly related to muscle microstructure, dMRI has been leveraged as a tool to assess how differences in dMRI measurements between muscles, genders, and athletes are predictive of muscle function over time, and in response to injury.

Human performance is a key area of interest where dMRI has been leveraged to assess microstructural changes associated with muscle function. There are three main categories of research that have been studied in the context of dMRI in human performance: 1) the relationship between dMRI measurements and muscle function; 2) assessment of acute microstructural insults to the muscle; and 3) long‐term microstructural adaptations to external stimuli. The overall goal of leveraging dMRI in the context of human performance is to better understand baseline function in an athlete, potential return to play, and how an athlete is responding to a training or rehabilitation routine.

### Relationship Between dMRI and Muscle Function

At baseline, for dMRI to be a useful tool for assessing performance, the sensitivity of dMRI metrics to whole muscle function (i.e., power, work), needs to be established. Several studies have attempted to correlate dMRI measurements to muscle performance, particularly in the lower leg. These studies generally utilize an isokinetic dynamometer to assess voluntary mechanical power or torque of leg muscles as a function of angular velocity,^68^, ^69^, ^70^ fatiguing exercise,^71^ or maximum voluntary contractions.^69^, ^72^ Generally, these studies have shown that MD, RD, and *λ*_3_ positively correlate, and FA negatively correlates to muscle function, explaining ~17%–30% of the variance in the model. For example, a study by Klupp et al demonstrated that dMRI metrics were better predictors of lumbar extension/flexion strength than paraspinal muscle volume or fat fraction in healthy volunteers.^73^ This finding is supported by work by Berry et al, in which the predictive capacity of lumbar muscle size, microstructure, and fatty infiltration on lumbar postures were investigated in active‐duty Marines with and without low back pain.^74^ FA was found to be the strongest predictor of lumbar posture in several different simulated operational positions, along with the absence of association between muscle volume and lumbar spine posture. These findings suggest that muscle microstructure, rather than gross morphology, is an important predictor of lumbar spine function.

Muscles are heterogeneous in nature, and may have multiple subcompartments that have different contributions to whole muscle function. Recently, several groups have demonstrated regional heterogeneity of muscle fiber microstructure. Hooijmans et al demonstrated that the biceps femoris and vastus medialis have increasing MD from distal to proximal regions,^75^ which in turn respond differently to exercise stimuli. Berry et al found regional differences in paraspinal muscle size and restricted diffusion in the upper and lower lumbar spinae that may be reflective of the roles of these muscles to stabilize and provide motion of the spinal column.^58^ Overall, these findings demonstrate that dMRI is sensitive to regional differences in microstructure within a muscle itself, which is important for understanding both muscle function at baseline and to increase sensitivity to microstructural changes in response to a local injury or therapy.

### Assessment of Microstructural Insults to Muscle

Skeletal muscle injuries are highly common in recreational and elite athletes. Under normal physiologic conditions, exercise‐induced muscle microtrauma is able to be effectively and efficiently repaired, resulting in the addition of myofibrils within a myofiber. However, if a muscle is exposed to repetitive microtrauma (i.e., running), a complex physiological healing cascade occurs, where microstructural changes in muscle are present for extended periods of time, resulting in alterations in muscle performance. Keller et al utilized dMRI and T2 mapping to study acute changes in muscle microstructure before and 3 hours after competition in a triathlon.^76^ Compared to baseline, increased MD, *λ*_3_, and *λ*_1_, and decreased FA were found in the hamstring muscles, suggesting microstructural damage of the myofibers due to exercise. Interestingly, no change in T2 relaxation—an MRI biomarker related to inflammation—was found. Similarly studies in marathon runners before, 1–2 days after, and 2–3 weeks after competition, increases in MD, *λ*_1_–*λ*_3_ were observed postmarathon, that returned to baseline values upon follow‐up.^77^ Changes between time points were more pronounced in the biceps femoris and the vastus medialis than in other muscles studied. Furthermore, changes between time points were more pronounced in different regions of the muscle, suggesting that changes in muscle microstructure in response to long‐distance running are not homogeneously distributed throughout the length of a muscle. In a study of adolescent elite rowers, differences in MD and FA were observed in the paraspinal muscles 6 hours after training between seep and scull style rowers, which is potentially due to greater eccentric contraction in sweep style rowing during the catch phase.^78^ Collectively, these findings shed light on the diverse regional and temporal aspects of muscle adaptation to acute exercise and recovery.

### Long‐Term Microstructural Adaptations to External Stimuli

dMRI enables the detection of subtle changes in muscle microstructure, which is a highly valuable metric for assessing effectiveness of physical therapy or training paradigms on muscle. Okamoto et al utilized dMRI in a hybrid training protocol consisting of electrically stimulated isometric muscle contractions to show increased diffusion metrics in leg muscles in patients with nonalcoholic fatty liver disease over a 6‐month training window.^79^ In a study evaluating the effect of Nordic and diver hamstring exercises on muscles over a 12‐week period, increases in fascicle length and orientation were observed, suggesting these exercises cause measurable architectural changes in muscle, which has implications for hamstring injury prevention.^80^ In a study evaluating the effect of isokinetic eccentric training on human shoulder strength, similar changes in fascicle length were observed, but no change in FA was observed (MD, RD, and *λ*_1_–*λ*_3_ were not reported).^81^ Lastly, muscle hypertrophy has been observed in people who routinely run for exercise in comparison to controls,^82^ which is likely driven by myofiber hypertrophy associated with increased RD and decreased FA.^83^

## Clinical Applications of DT‐MRI in Skeletal Muscle

### Muscular Dystrophies and Inflammatory Myopathies

Musculoskeletal diseases often present as diminished function or weakness during development. The diagnostic process typically includes a detailed clinical history followed by electrophysiological and genetic testing. Skeletal muscle biopsies may also be performed to obtain tissue samples for further analysis to confirm the diagnosis and to guide treatment planning.

**Figure 8:**
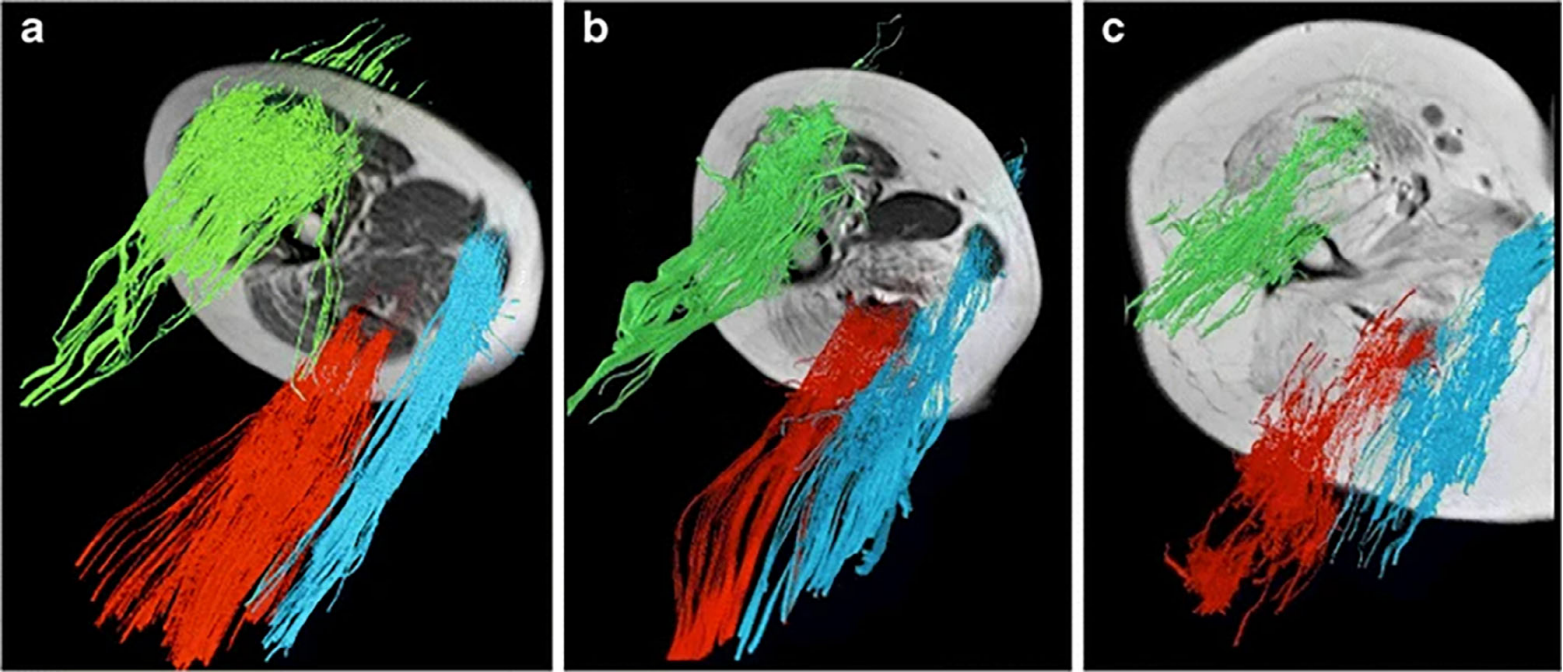
DTI tractography of rectus femoris (green), biceps femoris (red), and gracilis (blue) muscles in a (**a**) early ambulatory 6‐year‐old boy with Duchenne muscular dystrophy (DMD), (**b**) late ambulatory 8‐year‐old boy with DMD, and (**c**) early nonambulatory 12‐year‐old boy with DMD, superimposed on anatomical T1‐weighted images. These images demonstrate decreasing number, length and organization of fiber tracks with increasing disease severity. Figure reproduced with permission from Springer Link (2015).^57^

Quantitative MRI is currently being leveraged as a technique to noninvasively assess disease progression and potential response to treatment for a number of musculoskeletal diseases of interest. One of the most studied applications is muscular dystrophy, which can be broadly summarized as a group of genetic disorders that are characterized by progressive weakness and degeneration of muscles that are critical for movement and function. Duchenne muscular dystrophy (DMD) is the most common presentation of this disorder, and thus it was one of the early musculoskeletal diseases that was studied with dMRI. Ponrartana et al demonstrated that as DMD progressed and increased intramuscular fatty deposition was observed, there was a decreasing number of fiber tracks, decreased length of fiber tracks, and decreased organization of fiber tracks from tractography of patients with DMD (Fig. 8).^57^ FA was found to negatively correlate with Medical Research Council (MRC) score—a biomarker of function—and fat fraction.^57^ Hooijmans et al evaluated cofounders of pathophysiology on the ability to measure microstructural differences in the leg muscles of patients with DMD using a multiparametric MRI protocol.^84^ Using both experimental and simulation‐based^12^, ^35^ approaches, they demonstrate how confounding effects of both image quality (SNR) and tissue quality (fat fraction) can influence the ability to resolve tissue‐based differences in patients with DMD. Namely, increased fat fraction and low SNR result in underestimation of λ_1_–λ_3_ and overestimation of FA.^53^, ^84^

While most studies of DMD are performed with spin‐echo‐based dMRI pulse sequences, two studies have leveraged the long diffusion time capabilities of stimulated echo dMRI to evaluate differences in muscle microstructure in pathologic vs. healthy muscle. McDowell et al evaluated the effect size differences in dMRI measurements between healthy control and DMD patients over a range of Δ and found that a Δ of 190 msec resulted in the highest sensitivity between the two groups,^85^ which is supported by Berry et al.^18^ In a preclinical mouse model, Winters et al leveraged RPBM modeling to monitor age‐related changes in muscle microstructure in animals with and without MDX.^23^ Although this animal model does not present with the same relative fatty degeneration and dilated cardiomyopathy phenotype as what is observed in humans,^86^ this model does demonstrate increased fiber permeability and fibrosis. Increased variability was observed in RPBM measured output variables, which may be suggestive of the increased heterogeneity of tissue microstructures in this animal model.

Several studies have recently begun to leverage dMRI to investigate microstructural changes in skeletal muscle associated with Beckers muscular dystrophy (BMD). BMD is similar to DMD, except it is characterized by a milder presentation of symptoms, has a later onset, and generally a longer life expectancy. Using a spin echo dMRI technique, there have been mixed findings in identifying microstructural differences between BMD patients and controls. Hooijmans et al identified fatty replacement as the primary predictor of decreased functional capacity, with no difference in diffusion indices of nonfat replaced muscle in comparison to healthy controls.^86^ However, Maggi et al^87^ and Nava et al^88^ identified increased FA and lower MD/RD in BMD patients in comparison to controls. Interestingly, the variance in the diffusion indices was demonstrated to be a better predictor of disease state than the mean diffusion indices. This suggests that leveraging dMRI to detect increased microstructural heterogeneity observed with injury may be a better predictor than the mean changes in muscle microstructure. Finally, using a stimulated echo dMRI protocol in conjunction with RPBM model—albeit at only 3Δ—Cameron et al identified no diffusion‐based differences between controls and BMD patients.^26^ However, via RPBM modeling, there were significant differences in modeled estimates of muscle microstructure which was also observed histologically. However, in paired comparisons in five patients who received both dMRI and muscle biopsy, no significant association between mean or variance in fiber diameter was observed. Although, this relationship was likely underpowered, especially given difficulty in colocalization of muscle biopsy and dMRI measurements.

Polymyositis (PM) and dermatomyositis (DM) are autoimmune disorders characterized by muscle inflammation and weakness, with DM additionally involving skin rash.^89^ Diagnosis typically involves a combination of clinical assessment, muscle strength testing, blood tests to detect muscle enzyme levels, electromyography (EMG) to assess muscle electrical activity, and muscle biopsy to confirm the presence of inflammation. Several groups have sought to leverage dMRI to complement traditional diagnostic methods for these conditions. Studies have demonstrated that patients with PM and DM have increased baseline MD, RD, and decreased FA in comparison to controls^90^, ^91^ that is likely due to higher baseline levels of inflammation in the tissue.^20^ Furthermore, in response to exercise, there is an increased change in RD in patients with PM and DM compared to controls,^92^ potentially related to increased muscle fiber damage associated with inflammation.^93^ Although DM is fundamentally different from muscular dystrophy given standard anatomic imaging, they have similar presentations. Ran et al leveraged DTI and DKI to differentiate patients with DM from those with muscular dystrophy and identified that those with DM had increased overall diffusivity.^92^ They defined optimal cut‐off values for ADC and MD values between DM and muscular dystrophy given their multiparametric imaging protocol. Taken together, these studies point to a need to further standardize multiparametric imaging approaches to increase sensitivity to disease diagnosis, progression, and potential response to treatment.

### Age‐Related Muscle Changes

**Figure 9:**
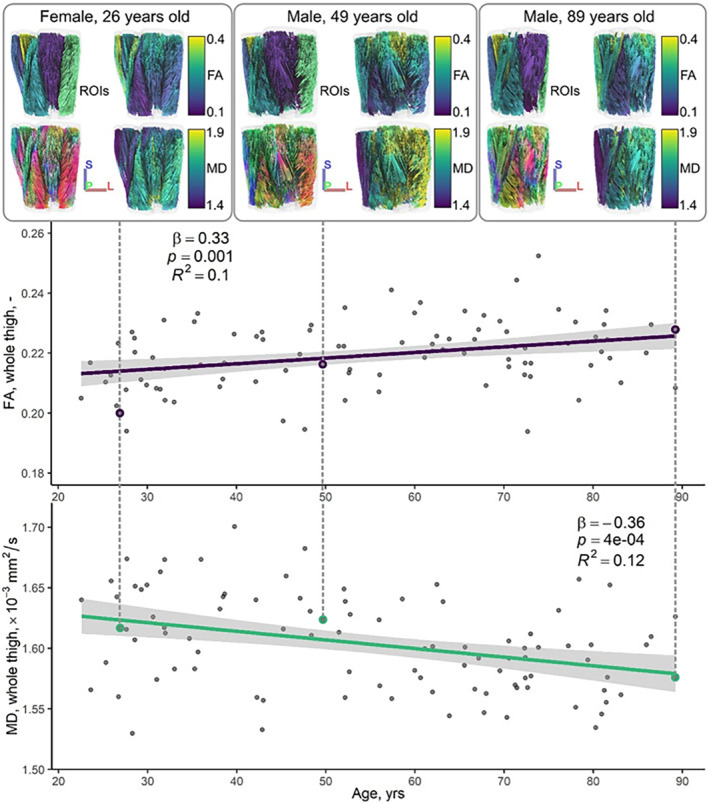
dMRI metrics and tractography as a function of age. Representative tractography results (top row), whole‐thigh fractional anisotropy (FA; middle row), and mean diffusivity (MD; bottom row) measures as a function of age. Dashed lines link the tractography examples to their corresponding FA and MD data points, which are highlighted by colored circles on the scatter plots. Tractography results are presented as anterior coronal views of the thigh showing, in clockwise order from the top left, muscle regions of interest, tracts colored by their average FA and MD, respectively, and fiber tract directionality (red, left–right; green, anterior–posterior; blue, superior–inferior). In the scatter plots, standardized linear regressions demonstrate associations between FA and age and MD and age, and regression lines, 95% confidence intervals, and regression statistics are shown. Figure reproduced with permission from John Wiley and Sons (2023).^69^

With advancing age, skeletal muscle undergoes various structural and functional changes collectively termed sarcopenia. These changes include whole muscle atrophy, a decrease in the number and size of muscle fibers, increased intramuscular fat deposition, and fibrosis. This results in concomitant changes in muscle function, architecture, and metabolism, resulting in a decline in muscle force‐generating capacity and endurance.^94^ Overall, age‐related changes in skeletal muscle contribute to decreased mobility, increased risk of falls, and compromised quality of life in older adults.^95^ dMRI has been leveraged by several groups to investigate microstructural changes associated with aging. Overall, there is some evidence that increased FA and decreased MD is associated with aging,^69^, ^96^, ^97^ which can be physiologically explained by normal processes of fiber atrophy. However, this is not consistently found across the literature,^98^ with the opposite trend of decreased FA and increased MD, which is hypothesized to be caused by a decrease in fiber asymmetry and an increase in permeability with age.^27^ A study by Cameron et al found increased FA and decreased MD associated with age, demonstrating about 10% of the variance in FA and 12% of variance in MD is associated with age (Fig. 9). In addition to reporting diffusion indices, tractography and functional muscle testing was additionally performed, with nonsarcomere length‐corrected PCSA from tractography serving as the best predictor of muscle strength, explaining 32% of the variance for biceps femoris longus and 33% of the variance in rectus femoris. Interestingly, while dMRI studies of skeletal muscle in aging have shown some effect size, the value in these studies is to identify subjects who are either biologically aging prematurely or have a high capacity to respond to training stimuli to reverse the natural cause of aging. However, dMRI on its own is unlikely to be a strong predictor of these health biomarkers, but can be paired with functional assessments of muscle activation such as IVIM^33^ or creatine chemical‐enhanced saturation transfer (CrCEST)^99^—a molecular imaging tool that can be leveraged to investigate muscle activation by spatially quantifying metabolites that involved in ATP synthesis—to enhance understanding of muscle functional capacity.

### Hamstring Injuries/Return to Sport

Hamstring injuries are common among athletes, in particular those involved in sports requiring sprinting, jumping, and sudden changes in direction. On a physiological scale, these injuries often involve overstretching or tearing of the hamstring muscles, typically occurring at the myotendinous junction.^95^ These injuries often cause pain, swelling, decreased mobility, and prevent participation in sport, and have an increased likelihood of reinjury. Thus, this has driven interest in utilizing dMRI to characterize hamstring injuries and potentially leverage this tool to predict return to sport. In a study of 41 athletes scanned within 7 days of injury, increased diffusivity was observed compared to control muscle; however, no differences in FA or T2 relaxation were observed.^100^ At 2 weeks after the initial visit, increased MD, *λ*_1_, and *λ*_2_ was observed compared to controls; however, by return to play, all diffusion metrics had returned to baseline. These findings are similar to those found in a similar study of division one athletes with a hamstring strain. At the time of injury, an increase in all diffusion metrics, but not FA, was observed; however, these metrics return to baseline by the return to play.^101^ One of the goals of this technique is to determine if dMRI indices at the time of injury can predict when an athlete can return to competition. Two studies from the same group found that using dMRI metrics alone were unable to predict the number of days needed for return to athletic competition.^102^ However, by leveraging radiomics to account for dMRI, T2‐weighted, and T1‐weighted data from the same patient, a model was developed that can predict return to competition.^103^ These studies suggest that a multiparametric MRI protocols are more likely to predict an athletes timeline for recovery after injury.

## Discussion

dMRI and its many associated analytical strategies are becoming widely used for musculoskeletal research. This technique is repeatable, can be extended to study any muscle in the body, and does not require any additional equipment.^104^, ^105^, ^106^, ^107^

**Figure 10:**
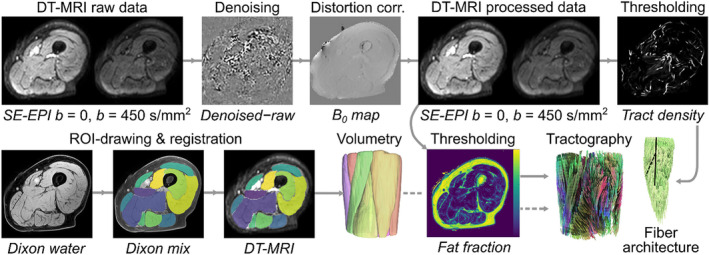
Schematic showing an example dMRI processing pipeline for a study. Representative axial spin‐echo dMRI and chemical‐shift‐based water‐fat separation images from the upper leg are shown for a 89‐year‐old male subject. The top row shows the dMRI raw data, which undergoes denoising, distortion correction, and calculation of the diffusion tensor. The bottom row shows region of interest (ROI) drawing on fat‐water images and registration of ROIs to the dMRI data. Tractography and fiber length/orientation was performed on the processed dMRI data using muscle volumes as seed regions. Figure reproduced with permission from John Wiley and Sons (2023).^69^

A key barrier to adoption of dMRI in the clinic is the establishment of baseline values that have significance to a practitioner and a patient. One of the reasons this has been difficult to implement is, currently, there is no standard dMRI protocol that has been widely adopted across many sites, nor a standardized postprocessing algorithm, which makes it difficult to compare results between studies. For example, while the minimum number of diffusion directions that needs to be sampled to calculate the DT is 6, increasing the number of diffusion directions increases accuracy in the calculation of the DT. Thus, there has been a trend in recent years with increasing number of studies collecting over 30 diffusion directions of data. In addition, Δ has a large effect on the DT, with increased Δ resulting in higher FA and lower MD/RD values. However, the majority of studies fail to report the Δ that was used. Furthermore, there are a number of processing pipelines that can be leveraged to analyze dMRI data. An example of how this pipeline may look can be found in Fig. 10. Lastly, muscle is an isovolumetric tissue. Given this and the problems associated with fiber cross‐sectional areas changing with changes in fiber length, standardizing the length of the muscle via joint position standardization would greatly aid in comparing dMRI measurements between studies of the same tissues. Although a standardized protocol for acquisition or analysis does not currently exist, in order to begin a dialogue toward standardization, a list of recommendations and tools for performing a dMRI experiment in skeletal muscle can be found in Table 2. Of note, there will be inherent variance between protocols due to differences in the scientific question that is being asked, the muscle that is being scanned, the age and size of the subject, the duration of the sequence, and hardware/software differences between vendors. However, this can serve as a preliminary effort toward standardization of dMRI acquisition and analysis.

**Table 2:** List of Recommendations for Acquisition and Analysis of dMRI Data for Skeletal Muscle

	Feature	Recommendation
Acquisition	Pulse sequence	Spin echo sPFG for general diffusion measurements
		Stimulated echo sPFG for long diffusion times (increased fiber size sensitivity)
		dPFG for high sensitivity to fiber size/heterogeneity
		OGSE for high temporal resolution
	Δ	Spin echo sPFG—20 msec
		Stimulated echo sPFG—170 msec^18^
	*b*‐value	400–500 seconds/mm^2^ (Froeling et al^12^)
	Diffusion directions	At least 12, 20–30 is better, 60 or greater for high fidelity measurements
	Voxel size	2 mm × 2 mm in plane
	Slice thickness	4 mm, isotropic is better
	Slice gap	None, especially for tractography
	Echo time	Minimize, <60 msec
	Repetition time	Allow full relaxation, ~2 seconds
	Fat suppression	Spectrally adiabatic inversion recovery (SPAIR) is most commonly used
	Joint positioning	180°: knee, elbow, hip, forearm. 90°: ankle, shoulder. Normal lordosis: spine
Analysis	Image registration	Rigid or b‐spline transformations
	Denoising	Principle component analysis^108^, ^109^
	Analysis software	AFNI, DTITools, FSL, DTI‐TK, MRTrix, Matlab, DiTSI (dPFG)
	Tissue modeling	RPBM—note, it requires stimulated echo sPFG data at multiple Δ
	ROI selection	Mimics, Slicer, Horos, ITK‐SNAP
	Quality control	Visually inspect MRI data at all steps in the data analysis pipeline
		Ensure consistent coverage of the entire muscle volume
	Tractography	DTITools, DSI Studio, Matlab, Diffusion Toolkit

sPFG, single‐pulsed field gradient; dPFG, double‐pulsed field gradient; OGSE, oscillating‐gradient spin‐echo; Δ, diffusion time; DOFS, Dixon olefinic fat suppression.

**Figure 11:**
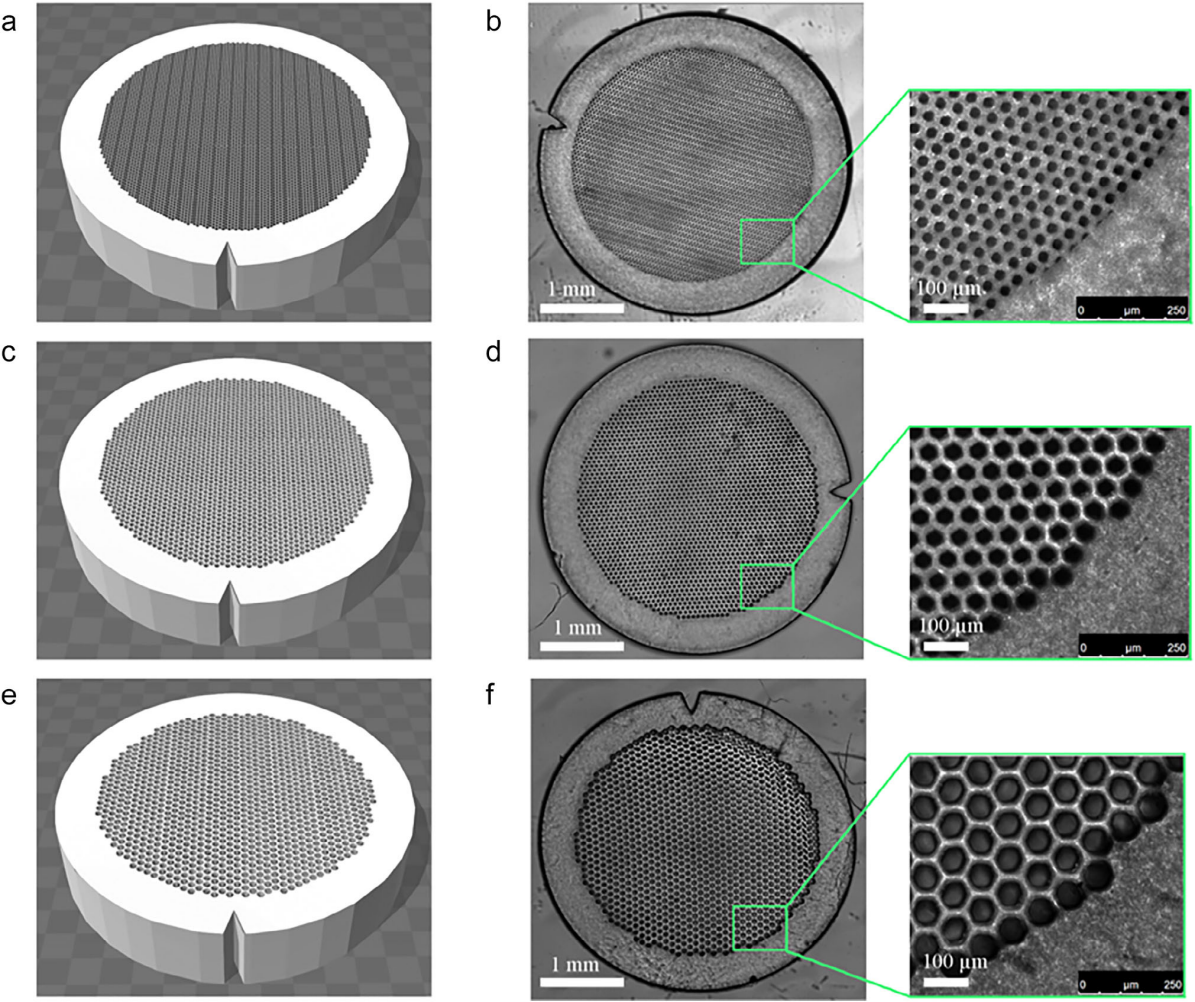
Example 3D computer‐aided design renderings (left column) and light‐based 3D printed phantoms (middle and right column). (**a**, **b**) A 30‐μm ideal geometry phantom. (**c**, **d**) A 50‐μm ideal geometry phantom. (**e**, **f**) A 70‐μm ideal geometry phantom. Figure reproduced with permission from Mary Ann Liebert (2017).^110^

To bring consensus between studies, there needs to be a critical evaluation of how pulse sequence parameters affect our ability to resolve specific features of muscle microstructure. One potential approach to reconcile the effect of different scanning parameters and analysis protocols between groups on resulting dMRI measurements is the establishment of a common phantom with tissue informed microstructure that can be scanned and analyzed at multiple locations. Techniques including light‐based 3D printing have been previously utilized to fabricate phantoms with idealized‐ and histology informed microstructure that replicate the restricted diffusion profile that is found in skeletal muscle (Fig. 11).^110^

Skeletal muscle is only part of a multitissue system involved in generating motion in the body. The majority of studies that have utilized dMRI to study skeletal muscle ignore other key components in the musculoskeletal system such as tendon and nerve. One of the key reasons tendons are not often studied is due to the ultrashort relaxation times of these tissues, making it highly difficult to gain any quantitative information from these tissues. However, techniques such as ultrashort echo time (UTE) can and should be leveraged in combination with dMRI in order to more closely study the myotendinous junction, especially in the context of injuries such as hamstring strain. For a recent, comprehensive review on tendon evaluation with UTE, see Malhi et al.^111^

Utilizing DT‐MRI in conjunction with other sequences, such as spectroscopy, T2 mapping, or fat‐water imaging, allows for a multiparametric approach that can improve our understanding of muscle tissue properties in the context of disease or injury. Muscle activation is controlled by motor neurons and thus neurologic impairments can have functional impairments on muscle, regardless of microstructure or architecture. MRI can provide spatial maps of sensitive information on regions of muscle that have activation impairments in a way that traditional assessments of muscle activation (eg, EMG) cannot. Techniques such as IVIM or CrCEST can be leveraged to changes in a muscle after muscle activation (i.e., exercise). IVIM has been used to demonstrate dose‐dependent responses to exercise in a muscle,^112^ and have even shown the ability to predict exercise‐based rehabilitation responses in individuals with low back pain.^113^ Assessment of changes in blood flow or energy‐related metabolites after exercise can yield information about muscle activation that is difficult to quantify using traditional assessments.

In conclusion, dMRI of skeletal muscle has become a widely available technique that is being leveraged to study a wide array of musculoskeletal disorders. Current innovation in pulse sequence development, analysis tools, and new applications in both preclinical animal and human models reflect the high value of noninvasive tools to assess muscle microstructure and architecture. Leveraging dMRI in conjunction with other MRI‐based tools provides an opportunity to have a better picture of whole muscle health, as muscle microstructure is only one component—albeit an important one—of muscle function. As these techniques are being developed, it is critical to have robust validation and description of data acquisition and analysis approaches to ensure repeatability of experiments across sites and between vendors.
